# Transgenic Expression of Nrf2 Induces a Pro-Reductive Stress and Adaptive Cardiac Remodeling in the Mouse

**DOI:** 10.3390/genes13091514

**Published:** 2022-08-24

**Authors:** Arun Jyothidasan, Sini Sunny, Saravanakumar Murugesan, Justin M. Quiles, Anil Kumar Challa, Brian Dalley, Senthil Kumar Cinghu, Vivek Nanda, Namakkal-Soorappan Rajasekaran

**Affiliations:** 1 Molecular and Cellular Pathology, Department of Pathology, University of Alabama at Birmingham, Birmingham, AL 35294, USA; 2 Division of Molecular and Translational Biomedicine, Department of Anesthesiology and Perioperative Medicine, University of Alabama at Birmingham, Birmingham, AL 35294, USA; 3 Department of Pharmacology, Skaggs School of Pharmacy and Pharmaceutical Sciences, University of California, San Diego, CA 92093, USA; 4 Department of Biology, University of Alabama at Birmingham, Birmingham, AL 35294, USA; 5 Huntsman Cancer Center-Genomic Core Facility, University of Utah, Salt Lake City, UT 84112, USA; 6 Epigenetics & Stem Cell Biology Laboratory, National Institute of Environmental Health Sciences, National Institutes of Health, Research Triangle Park, NC 27709, USA; 7 Department of Medicine, University of Utah School of Medicine, Salt Lake City, UT 84132, USA; 8 Center for Free Radical Biology (CFRB), University of Alabama at Birmingham, Birmingham, AL 35294, USA

**Keywords:** Nrf2 transgene, constitutively active Nrf2, RNAseq, echocardiography, reductive stress

## Abstract

Nuclear factor, erythroid 2 like 2 (Nfe2l2 or Nrf2), is a transcription factor that protects cells by maintaining a homeostatic redox state during stress. The constitutive expression of Nrf2 (CaNrf2-TG) was previously shown to be pathological to the heart over time. We tested a hypothesis that the cardiac-specific expression of full length Nrf2 (mNrf2-TG) would moderately increase the basal antioxidant defense, triggering a pro-reductive environment leading to adaptive cardiac remodeling. Transgenic and non-transgenic (NTG) mice at 7–8 months of age were used to analyze the myocardial transcriptome, structure, and function. Next generation sequencing (NGS) for RNA profiling and qPCR-based validation of the NGS data, myocardial redox levels, and imaging (echocardiography) were performed. Transcriptomic analysis revealed that out of 14,665 identified mRNAs, 680 were differently expressed (DEG) in TG hearts. Of 680 DEGs, 429 were upregulated and 251 were downregulated significantly (FC > 2.0, *p* < 0.05). Gene set enrichment analysis revealed that the top altered pathways were (a) Nrf2 signaling, (b) glutathione metabolism and (c) ROS scavenging. A comparative analysis of the glutathione redox state in the hearts demonstrated significant differences between pro-reductive vs. hyper-reductive conditions (233 ± 36.7 and 380 ± 68.7 vs. 139 ± 8.6 µM/mg protein in mNrf2-TG and CaNrf2-TG vs. NTG). Genes involved in fetal development, hypertrophy, cytoskeletal rearrangement, histone deacetylases (HDACs), and GATA transcription factors were moderately increased in mNrf2-TG compared to CaNrf2-TG. Non-invasive echocardiography analysis revealed an increase in systolic function (ejection fraction) in mNrf2-TG, suggesting an adaptation, as opposed to pathological remodeling in CaNrf2-TG mice experiencing a hyper-reductive stress, leading to reduced survival (40% at 60 weeks). The effects of excess Nrf2-driven antioxidant transcriptome revealed a pro-reductive condition in the myocardium leading to an adaptive cardiac remodeling. While pre-conditioning the myocardial redox with excess antioxidants (i.e., pro-reductive state) could be beneficial against oxidative stress, a chronic pro-reductive environment in the myocardium might transition the adaptation to pathological remodeling.

## 1. Introduction

Nrf2 (Nfe2l2) is a transcription factor that primarily responds to oxidative damage by enhancing the transcription of antioxidant genes [1]. Nrf2 also regulates other genes involved in inflammation [2], autophagy [3] and proteostasis [4], demonstrating its pleiotropic role. Under basal conditions, Nrf2 binds to its repressor, Keap1 and is constantly degraded in the cytoplasm [5]. In response to stress (i.e., oxidants, toxic insults, electrophiles), the sensitive cysteine residues in Keap1 undergo conformational changes resulting in the dissociation of Nrf2, which then translocates into the nucleus and binds to the antioxidant response element (ARE) of target genes [1,5]. These genes encode for phase II xenobiotic-metabolizing enzymes, antioxidant proteins, non-enzymatic antioxidants (e.g., glutathione), DNA repairing enzymes [6,7,8], anti-inflammatory proteins [9], and molecular chaperones [10], and have AREs in their promoters.

Despite the cytoprotective role, the genetic deletion of Nrf2 has little or no impact on the survival of Nrf2 knockout (Nrf2^–/–^) mice under unstressed conditions [11,12,13]. However, in the case of Nrf2 deletion accompanied with physiological stress (exercise), we and others reported that the surge in ROS increases susceptibility of the myocardium to oxidative stress and cardiac dysfunction ultimately impacting survival [14,15,16,17,18]. In contrast, sustained activation of Nrf2 and the transactivation of ARE-containing genes in the absence of stress could be detrimental as it causes reductive stress in the myocardium [19]. While constitutively active Nrf2 (CaNrf2) was shown to be detrimental through excessive transcription of ARE-genes and inducing RS, here, we investigated whether enhancing the Nrf2 expression could increase basal antioxidant defense without triggering pathology in the heart. Therefore, in contrast to unrestricted Nrf2 signaling during CaNr2-mediated reductive stress, we hypothesize that the transgenic overexpression of full-length Nrf2 promotes an appropriate level of antioxidant induction to promote the basal defense mechanisms in the heart. Indeed, next generation sequencing (NGS) analysis revealed that over 680 genes are differentially regulated and the majority of them are found to be involved in ROS metabolism, glutathione conjugation and Nrf2 signaling. There was a mild increase in the hypertrophic gene expression and echocardiography analysis indicating a slightly increased systolic function in the TG mice. These observations support that the readily available antioxidant abundance may render protection against toxic insults including oxidative stress.

## 2. Methods

### 2.1. Transgenic mNrf2-TG (Full-Length Nrf2) Mouse and Genotyping

Transgenic mice with cardiac-specific expression of Nrf2 (mNrf2-TG) in C57/Bl6J background and non-transgenic litter mates at 6–7 months of age were used in this study [20]. Mice were housed under controlled temperature and humidity, a 12 h light/dark cycle, and fed with a standard rodent diet and water ad libitum. The Institutional Animal Care and Use Committee (IACUC) at the University of Alabama at Birmingham and the University of Utah, Salt Lake City, Utah approved all animal experiments, in accordance with the standards established by the US Animal Welfare Act. Tails were clipped (~1 mm) and genomic DNA from mNrf2-TG and controls were isolated using Hot Shot lysis method. Custom-designed mNrf2-TG primer pair sequences were used for Genotype validation (Table S4A). PCR was performed in Bio-Rad Thermal cycler T100 using MyTaq Red Mix polymerase (BIO-25043) and on 1.5% agarose gel electrophoresis.

### 2.2. Next Generation RNA Sequencing

Purity of the isolated total RNA was confirmed using bio-analyzer and intact poly(A) transcripts were generated from total RNA, oligo(dT) magnetic beads and mRNA sequencing libraries using the TruSeq Stranded mRNA Library Preparation Kit (Illumina, San Diego, CA, USA, RS-122-2101, RS-122-2102). Purified libraries were qualified using a D1000 ScreenTape assay (Agilent, 5067-5582/3) with the 2200 TapeStation Instrument (Agilent Technologies, Santa Clara, CA, USA). cBot and TruSeq SR Cluster Kit (Illumina, Cat. GD-401-3001) were used to apply 18 pM of the sequencing library to a TruSeq v3 flowcell (Illumina) and for clonal amplification, respectively. Finally, the flow cell was transferred to the HiSeq. 2000 instrument and run on a 50-cycle single read sequence with TruSeq SBS Kit v3-HS reagents (Illumina, Cat. FC-401-3002). Novoindex (2.8) was used to create a reference index on a combination of the hg19 chromosome and splice junction sequences (USeq; v8.6.4, Huntsman Cancer Institute, Utah Bioinformatics Shared Resource Center) to make transcriptomes using Ensembl transcript annotations (build 67). Reads were aligned to the transcriptome reference index as described above using Novoalign (v2.08.01), allowing up to 50 alignments for each read. Useq’s SamTranscriptomeParser application was used to select the best alignment for each read and convert the coordinates of reads aligning to splices back to genomic space.

### 2.3. RNA Isolation, RT-qPCR, and Western Blotting

Approximately 15 mg of flash frozen cardiac tissue was thawed from mNrf2-TG and controls, then tissue was mechanically homogenized with 50 μL 2-Mercaptoethanol. Total RNA was isolated using RNeasy mini kit (Qiagen Inc., Valencia, CA, USA, Cat#74106), its quantity/quality were assessed by NanoDrop OneC Spectrophotometer (Thermo Fisher Scientific, Wilmington, DE, USA). The cDNA was synthesized from 1.25 μg of total RNA using QuantiTect reverse transcription kit (Qiagen, cat. no 205313). Quantitative RT-qPCR was performed using 25–50 ng cDNA of each sample and 1.0 pmol of respective primers in a 10 μL SYBR green reaction mix (Qiagen, 204056) and amplified in a Roche Light Cycler 480 (Roche, Basel, Switzerland). Relative expression was quantified using Ct values, and mRNA expression (fold-change) was calculated by normalizing to the Ct of stable genes *Gapdh* or *Arbp1* according to the 2^−ΔΔCt^ method.

For Western blotting, heart tissues from NTG, and mNrf2-TG (*n* = 3) at 6 months of age were mechanically homogenized in cytosolic extraction buffer and centrifuged at 5000 rpm for 6 min. The protein extract from all samples were collected, normalized, and resolved on 10% SDS-PAGE. The gel was blotted on PVDF membrane and blocked with TBST buffer containing 5% non-fat dry milk. Primary antibodies (Table S4B) were incubated overnight at 4C. Secondary antibody was incubated for 1 h at room temperature using horseradish peroxidase IgG (Vector Laboratories, Burlingame, CA, USA). Membranes were finally treated with ECL (Pierce, Rockford, IL, USA) and imaged using Amersham Imager 600 (GE Healthcare Life Sciences, Chicago, IL, USA).

### 2.4. Pathway Analysis

The transcriptome was mapped to mm10 (GRCm38 genome assembly). Deseq2 (Ver 1.32.0) package was used for normalization, visualization, and differential analysis of high dimensional count data. Gene list was then filtered to remove transcripts with low expression (aggregate sum of count across rows > 1). The transcriptome was left with 14,665 unique transcripts including predicted genes. From this transcriptome, mRNA transcripts that had statistically significant changes between mNrf2-TG and control hearts were filtered using Deseq2 computed *p*-value (*p* < 0.05), which excluded 10,725 transcripts. All downstream analyses including PCA plots and heat maps were created in RStudio (Ver 1.4.1717). Gene set enrichment analysis (GSEA) was performed to determine whether the a priori defined set of genes showed statistically significant and concordant differences between transgenic and control samples. The datasets were loaded into GSEA and run with default parameters. All molecular interaction networks, biological pathways and integrating these networks with annotations, gene expression profiles was carried out in Cytoscape (Institute for Systems Biology, Seattle, WA, USA, Ver 3.8.2).

### 2.5. Statistics

Meta-analysis of the first two principal components PC1 and PC2 accounted for 56.85% and 15.78% of the total variation, respectively (Figure S1A,B). The third principal component PC3 was included to increase the percentage of explained variances to more than 80%. Log2FC was generated from deseq2 package. Relative increase or decrease in mNRf2-TG RNA expression with respect to control mice was expressed as Log2FC (Log2 Fold change), ranging from positive (upregulation) to negative (downregulation) values. Absolute fold change can be calculated using the following formulas: 2^Log2FC for positive values and (1/2)^Log2FC for negative values. Values for relative gene expression or cardiac functional parameters were expressed as mean ± SD for *n* = 4 in each experimental group. Statistics between NTG and mNrf2-Tg groups were performed with student’s t-test. Pairwise comparisons between NTG, mNrf2-TG and CaNrf2 were performed with one-way ANOVA in GraphPad Prism. Statistical significance was defined as * *p* < 0.05, ** *p* < 0.01, *** *p* < 0.001.

### 2.6. Echocardiography

Two-dimensional echocardiography acquisitions were performed in NTG and mNrf2-TG mice using Vevo2100 Imaging System (Fujifilm VisualSonics, Inc., Toronto, ON, Canada) equipped with 38 MHz mechanical transducer. Animals (*n*  =  6–8) were anesthetized under 1–2% isoflurane, supplemented with 100% oxygen. Heart rate, ECG signals and respiration were recorded. Parasternal long-axis-M mode images were analyzed for cardiac function using Vevo lab 3.1 software [21,22,23].

## 3. Results

### 3.1. Genetic Characterization of Cardiac-Specific Nrf2 Mice (mNrf2-TG)

**Figure 1:**
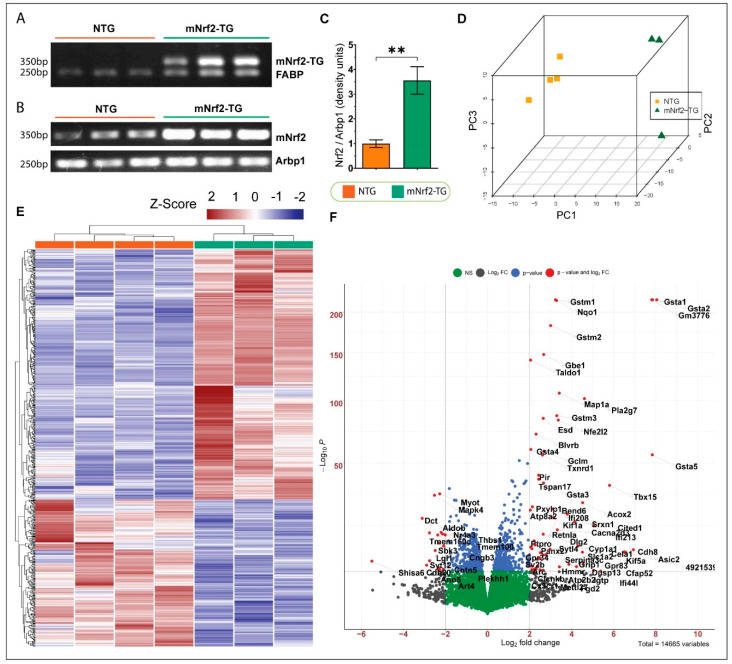
Heart specific Nrf2-Transgenic mouse model and transcriptomic changes. (**A**) Genotyping PCR for mNrf2-TG using αMHC forward and Nrf2 reverse primer (350 bp), (below) FABP (internal control) showed bands at 250 bp. (**B**) Semi-quantitative PCR for Nrf2 using primer for endogenous Nrf2 showing increased Nrf2 expression in Nrf2-TG compared to NTG mice; acidic ribosomal phosphoprotein (Arbp1/Rplp0) was used as housekeeping. (**C**) Quantification of semi-quantitative PCR for Nrf2 using image. (**D**) Three-dimensional principal component analysis for mNrf2-TG and NTG mice. (**E**) Heatmap representing expression of the 680 most significant (*p* < 0.05) differentially expressed genes (Log2FC > 1). Some 428 genes were upregulated, and 251 genes were downregulated in mNrf2-TG mice. The heatmap represents normalized count values (RLog) of each DEG in the individual samples, and genes were clustered unsupervised. Fold change > 2 and *p*-value < 0.05. (**F**) Volcano plot of DEGs showing log2fold change versus −log10 of (*p*-value) of transcripts identified by RNASeq analysis between NTG and mNrf2-TG. Significant DEGs expressed in mNrf2-TG (Log2FC ≥ 2 and *p* < 0.05) are highlighted in red (FDR corrected *p*-value). Blue dots indicate transcripts with a significant *p*-value but Log2FC ≤ 2; Grey dots indicate transcripts with a Log2FC ≥ 2 but with an FDR corrected *p*-value of <0.05; whilst green dots indicate non-significant transcripts with a Log2FC ≤ 2. The total number of genes amounts to 14,665. Error bars represent SEM. ** *p*-value < 0.01.

The transgenic (TG) mice were generated as described earlier [20]. The genotype of the TG mice was confirmed using the αMHC promoter sense and wild type Nrf2 antisense primer pair (350 bp; Figure 1A). An endogenous Nrf2 primers pair was designed to quantify both transgenic and wild type Nrf2 expression (250 bp; Figure 1B). Semi-quantitative PCR showed 3.5-fold increase in Nrf2 expression compared to NTG littermates at 6 months of age. (Figure 1C). Fatty acid-binding protein (*Fabp*) primers were used as quality control for genomic DNA, and acidic ribosomal phosphoprotein (*Arbp1* or *Rplp0*) was used for normalization.

### 3.2. Overall Outcomes of Myocardial Transcriptome in mNrf2-Transgenic Mice

Cardiac tissues from TG and NTG mice were used for NGS-based RNA sequencing. Principal component analysis (PCA) demonstrated a strong clustering of TGs vs. NTGs independently, suggesting unique changes in the myocardial transcriptome due to mNrf2 expression (Figure 1D). As shown in the heatmap, mNrf2-TG expression in the mouse hearts resulted in 680 differentially expressed genes (DEGs) (Log2FC > 1, i.e., fold change > 2 and *p* < 0.05). Among these, we observed 429 were upregulated (60%) and 251 (40%) were downregulated (Figure 1E). Moreover, the unsupervised hierarchical clustering of genes further validated the changes in the myocardial transcriptome of TG mice (Figure 1E). A volcano plot was generated to illustrate the top differentially expressed mRNAs with a threshold set at user-defined significance (i.e., *p* < 0.0001) with log fold change, i.e., Log2FC > 2 (i.e., fold change > 4.0) (Figure 1F).

### 3.3. Major Redox Pathways Are Activated in mNrf2-TG Cardiac Tissue

**Figure 2:**
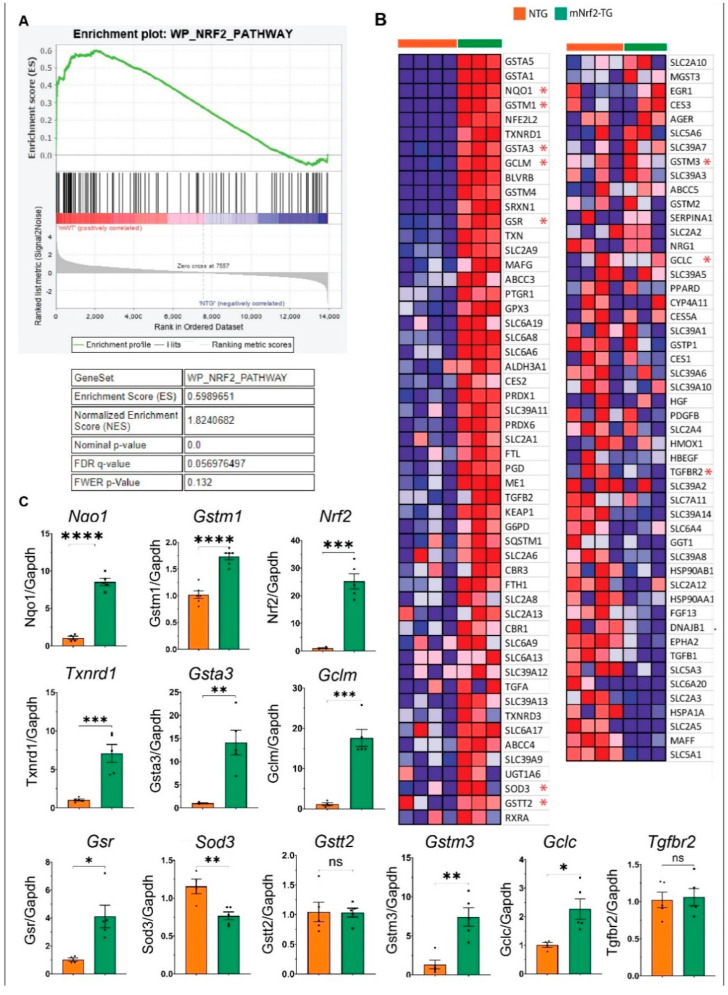
Antioxidant transcriptome in enhanced cardiac Nrf2 signalling. (**A**) GSEA plot of most enriched Nrf2 pathway from Wikipathways. GSEA algorithm calculates an enrichment score for a pathway by walking down the ranked list of genes, increasing a running-sum statistic when a gene is in the gene set and decreasing it when it is not. The magnitude of the increment depends on the correlation of the gene with the phenotype. The ES is the maximum deviation from zero encountered in walking the list. A positive ES indicates gene set enrichment at the top of the ranked list; a negative ES indicates gene set enrichment at the bottom of the ranked list. (**B**) Heatmap illustrating changes in 103 genes enriched in the Nrf2 pathway. (**C**) RT-qPCR validation of gene expression of *Gstm1*, *Gsta3*, *Gstt2 and Gstm3*, *Nrf2*, *Txnrd1*, *Sod3* and *Tgfbr2*. All RT-qPCR gene expression were normalized to Gapdh. Error bars represent SEM. * *p*-value < 0.05, ** *p*-value < 0.01, *** *p*-value < 0.001, **** *p*-value < 0.0001.

The gene set enrichment analysis (GSEA) of mNrf2-TG transcriptome (14,665 unique transcripts) clearly identified redox pathways as the most enriched pathways in Hallmark, Reactome and Wikipathways (Figure 2A and Figure S2A). The Nrf2 network from Wikipathways (WP, ES 0.59) enriched 103 genes, demonstrating changes in antioxidant genes are directly mediated by the transcriptional activation of Nrf2. Specifically, small *Maf* proteins which are co-factors of Nrf2, were downregulated in mNrf2-TG heart (*Maff* Log2FC-1.4; *Mafg* Log2FC 0.8), but *Mafk* [Log2FC − 0.1] was unchanged. Remarkably, other Nrf2 regulators displayed little or no change (*C-jun*, *Crebbp*, *Eif2ak3*, *Keap1*, *and Ubc*). Genes encoding for heat shock proteins (*Hspa1a*, *Dnajb1*, *Hsp90aa1*, *and Hsp90ab1*) were downregulated (Figure 2B and Table S1).

Members of the cytosolic glutathione transferases, *Gst*α and *Gst* mu, were significantly upregulated in mNrf2-TG hearts. *Gst* mu class members including *Gstm1*, *Gstm2*, *Gstm3*, *Gstm4* were upregulated while *Gstm5* and *Gstm6* were unchanged. Increased *Gstm1* and *Gstm3* expressions were subsequently validated in qPCR experiments (Figure 2C). All members of Gstα class (*Gsta1*, *Gsta2*, *Gsta3*, *Gsta4*, *and Gsta5*) were significantly upregulated (Figure 2B). However, neither *Gst* theta (*Gstt1*, *Gstt2*, *Gstt3*) nor *Gst pi* (*Gstp1*, *Gstp2*) class members showed any response to enhanced Nrf2 expression, confirming tissue specificity of one of its various isoforms [24]. Further, we confirmed the unchanged *Gstt2* expression using qPCR experiments (Figure 2C). Among the three microsomal glutathione S-transferases, *Mgst1* was most upregulated in TG (log2FC 1.4) and *Mgst3* was unchanged despite its abundant expression in cardiac tissue, identified through RNAseq. *Gst Kappa* (*Gstk1*, Log2FC − 0.7) was the only class of GSH transferases that was downregulated with increased Nrf2.

**Figure 3:**
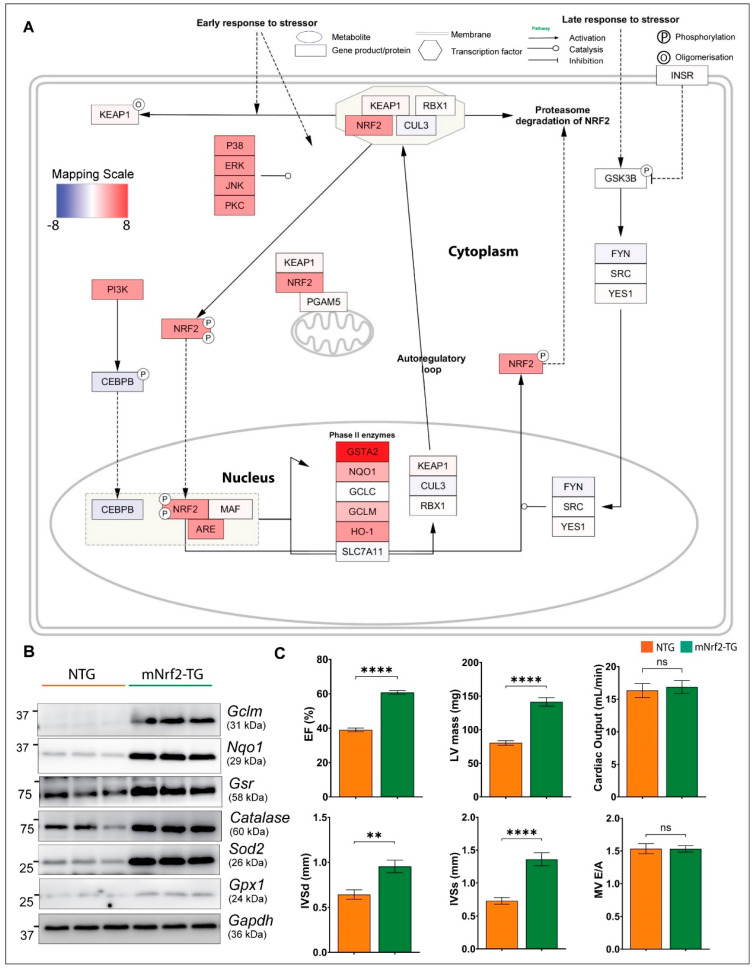
Gene expression mapped on the Nrf2 pathway (Wikipathways ID = 4357), cardiac structure and function. (**A**) Binding with Keap1/Cul3/RBX1 complex allows Nrf2 protein to be continually degraded via ubiquitination/proteosomal degradation. In response to ROS, Keap1 is oxidized or Nrf2 is phosphorylated resulting in translocation to nucleus. Pathway diagram shows changes in mNrf2-TG ARE targets when activated. Colour gradient scaled from blue (negative fold change) to red (positive fold change) was calculated using Log2foldchange, scaled and mapped onto curated network. Nodes (rectangle) represent genes or proteins and edges (or lines) represent interactions and relationships between connected nodes. (**B**) Validation of protein expression using Western blot, confirming the summary of changes in network. (**C**) Cardiac structure and function in mNrf2-TG mice. Two-dimensional non-invasive echocardiography (PSLAX axis) was used to determine systolic functions (i.e., ejection fraction and cardiac output), Left ventricle dimensions (i.e., LV mass, intraventricular septum diameter-systole and diastole) and diastolic function, i.e., mitral valve (early, E to late, A ratio) filling (*n* = 6–8/group).

Transcription factor *Junb* (log2FC − 0.6) was downregulated in mNrf2-TG hearts. Genes coding for major antioxidant enzymes including *Catalase*, *Gclc*, *Gclm*, *Gpx3 and Gpx4* were upregulated while no change was observed in Gpx1 (Figure S2A,B). Specifically, we confirmed that transcriptional induction resulted in increased protein levels for GCLM, NQO1, GSR, CATALASE, SOD2 and GPX1 using immunoblotting (Figure 3B). *Blvrb*, a gene encoding biliverdin reductase, a reducing enzyme that converts biliverdin to bilirubin (potent antioxidant), was significantly increased (Log2FC 2.3) in TG heart (Figure 2C). Similar to Wikipathways, Reactome identified glutathione conjugation pathway (Reactome, ES 0.8) (Table S3) and Hallmark (hallmark, ES 0.626) identified reactive oxygen species (ROS) as the top enriched pathway (Table S2).

### 3.4. Enhanced Keap1-Nrf2 Signaling Promotes Transcription of Downstream Targets of Nrf2

Many of the 680 DEGs appear to be direct targets of Nrf2-ARE signaling as the top upregulated gene code for antioxidants and/or other proteins implicated in ROS metabolism (Figure 2B and Figure S2B). The highest expression was recorded for glutathione transferases, *Gsta2* (Log2FC 8.04) and *Gsta1*(Log2FC 7.84), which may constitute up to 10% of cytosolic protein content [25]. Other top upregulated ARE targets of Nrf2 were reducing enzymes (reductases) such as *Gsr* (Glutathione reductase), *Nqo1* (superoxide reductase), *Txnrd1* (thioredoxin reductase) and *Srxn1* (cysteine sulfinic acid reductase). In addition, we observed significant changes in the transcription of Nrf2 regulators in mNrf2-TG hearts including the *Keap1* gene [Log2FC 0.48], suggesting a transcriptional induction of Keap1 to meet its need to repress excess Nrf2 (Figure 2B), while *Rbx1* was unchanged [Log2FC 0.12]. Another co-regulator of Nrf2, *Cul3* [Log2FC − 0.56], was downregulated significantly (Figure 3A).

### 3.5. Impact of Nrf2 on the Transcription of Selenoproteins (Thioredoxins and Peroxiredoxins)

Selenoproteins are one of three classes of antioxidants containing a selenocysteine (Se-Cys) amino acid residue [26]. *Txn1* (Log2FC 1.2), *Txnrd1* (Log2FC 2.6) *and Srxn1* (Log2FC 3.8) were upregulated in the hearts of mNrf2-TG mice. *Txnrd1* expression was validated using qPCR (Figure 2C). Likewise, various isoforms of peroxiredoxins (*Prdx 1–6*) were upregulated, except for *Prdx2*. Prdx1 (Log2FC 0.6) and *Prdx6* (Log2FC 0.6) were most upregulated in mNrf2-TG mice (Figure 2B). While glutathione peroxidase 1 (*Gpx1*) had no change in expression, *Gpx3* (Log2FC 0.77) and *Gpx4* (Log2FC 0.6) were upregulated in response to mNrf2. These notable changes demonstrate the extent of Nrf2 influence on the redox transcriptome in the hearts of transgenic mice.

### 3.6. Impact of Pro-Reductive Stress on Cardiac Structure and Function in mNrf2-TG Mice

With a persistent pro-reductive environment, the structure and function of the mNrf2-TG hearts were moderately altered without severe pathology. Echocardiography analysis revealed a modest increase in ejection fraction (EF) in mNrf2-TG compared to NTG mice (58 vs. 40%), while CaNrf2-TG hearts had a higher EF (~80%) indicating abnormally increased systolic function (Figure 3C). We previously demonstrated that the CaNrf2-TG mice exhibited LV hypertrophy, decreased chamber volume, and increased cardiac contractility at 6 months of age [17]. While we noticed an increase in LV mass, IVSd and IVSs, there was no change in cardiac output and diastolic function–mitral valve flow (MV E/A ratio) in mNrf2-TG mice (Figure 3C). These observations indicate that mNrf2-TG hearts with a pro-reductive condition are gradually adapting without severe pathology at 6 months.

### 3.7. Pro-Reductive Redox Promotes Adaptive Myocardial Remodeling without Influencing the Survival of mNrf2-TG Mice

In our previous study, we demonstrated that cardiac-specific constitutive active Nrf2 (truncated Nrf2/CaNrf2-TG) resulted in hyper-reductive stress, leading to pathological cardiac remodeling (hypertrophy) and significant mortality [19,27].

**Figure 4:**
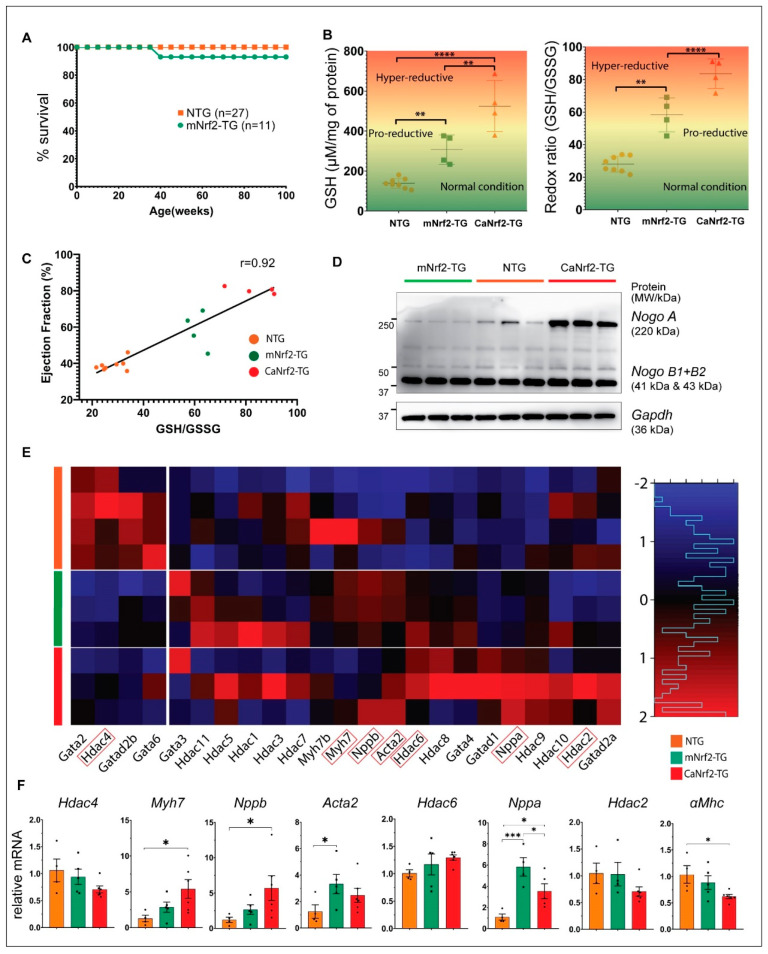
Survival, redox state, cardiac function, and remodelling in mNrf2 and CaNrf2 mice. (**A**) Kaplan–Meier survival analysis expressed in terms of percentage survival. (**B**) Redox scores of mNrf2-TG and CaNrf2 mice compared to controls, mapped on a gradient scale. (**C**) Redox state and cardiac function of NTG (orange) and mNrf2-TG (green) and CaNrf2 (red) hearts expressed in terms of ejection fraction (%) versus mean redox state (GSH/GSSG). EF and GSH data were paired within experimental groups. (**D**) Expression of *Nogo A* and *Nogo B* detected using Western blot from heart lysates. Membrane was stripped and reprobed for *Gapdh.* (**E**) RNAseq mRNA expression heatmap of common cellular hypertrophy indicators in normal, pro-reductive and hyper-reductive hearts. Log normalized data of genes scaled using R scale function shows distinctive changes between control, mNrf2-TG and CaNrf2. Column break for 4 markers (genes) that are downregulated in transgenic models together. Row breaks for groups with *n* = 3/4. (**F**) qPCR quantification of mRNA expression for cellular hypertrophy markers normalized to Gapdh. Error bars represent SEM.* *p*-value < 0.05, ** *p*-value < 0.01, *** *p*-value < 0.001, **** *p*-value < 0.0001.

Therefore, here we assessed the effect of the full-length mouse Nrf2 (mNrf2-Transgene)-driven pro-reductive state on cardiac health and survival (Figure 4A). While the CaNrf2-TG mice with hyper-reductive hearts showed significant mortality (>50%) at the age of ~60 weeks, 90% of the mNrf2-TG mice survived even at 80 weeks (Figure 4A). The ratio of oxidized-to-reduced glutathione, i.e., GSH/GSSG is often used as one of the measures of intracellular redox milieu [28]. A comparative analysis of the glutathione redox state in the hearts demonstrated significant differences between the pro-reductive vs. hyper-reductive conditions (139 ± 8.6; 233 ± 36.7 and 380 ± 68.7 µM/mg protein in NTG; mNrf2-TG and CaNrf2-TG) (Figure 4B). Similarly, the myocardial glutathione redox ratio ranges ~28–32 in NTG, but the mNr2-TG and CaNrf2-TG mice exhibited ~50–60 and ~80–90, respectively, suggesting a clear distinction in the myocardial redox state. The correlation between EF and glutathione redox state (GSH/GSSG) revealed a distinct distribution of NTG, mNrf2-TG and CaNrf2 (Figure 4C) with progressive cardiac remodeling in response to a steadily increasing reductive environment.

*Nogo A* expression has been demonstrated to play an important role in cardiac development and has been suggested as a potential indicator for heart failure [29]. Here, cardiac expression of *Nogo* (RTN4/Reticulon 4) was found to be high during hyper-reductive stress (CaNrf2-TG), whereas it was comparatively unexpressed in pro-reductive conditions (mNrf2-TG) (Figure 4D). However, *Nogo B1* and *B2* remained unchanged in all groups as it was shown to be primarily involved in inflammatory response, suggesting an absence of inflammatory phenotype in both CaNrf2-TG and mNrf2-TG hearts [30].

Adaptive or abnormal structural changes (i.e., hypertrophy) in the heart involve the reactivation of fetal cardiac genes in matured cardiomyocytes [31]. Dose-dependent changes in the mRNA levels of atrial natriuretic protein (*Anp/Nppa*), B-type natriuretic protein (*Bnp/Nppb*), α/β-myosin heavy chain (*αMhc*, *βMhc/Myh7*), and smooth muscle alpha (α)-2 (*Acta2*) were noticed in the TG mice (Figure 4E). Validation of the NGS data for these genes by qPCR showed similar dose-dependent upregulation of *Bnp* (2.5 vs. 5.0-fold), *βMhc* (2.5 vs. 5.0-fold) and decrease in *αMhc* (0.8 vs. 0.6-fold) in mNrf2-TG vs. CaNrf2-TG mice (Figure 4E). However, *Nppa* showed a remarkable increase in mNrf2-TG when compared to CaNrf2-TG (6 vs. 3.5-fold) mice (Figure 4E,F). Interestingly, both groups had a similar increase in *Acta2*, which encodes actin to stabilize the cytoskeletal structure of the cardiomyocytes (Figure 4E,F). In addition, GATA-binding factors (GATAs) and histone deacetylases (HDACs), the vital regulators of gene expression in cardiac myocytes were significantly altered in TG mice [32,33]. While most of the *Gata* isoforms were upregulated, Gata2 and Gata6 were decreased in both the TG mice (Figure 4E,F). Although Nrf2-mediated priming of the genes that regulate myocardial defense promotes a pro-reductive condition, this is inadequate to induce pathological hypertrophy or mortality through severe cardiac remodeling.

## 4. Discussions

Redox homeostasis is crucial for the dynamic function of cardiomyocytes as both oxidative and reductive stresses lead to cardiac pathophysiology [10,34,35,36,37]. Numerous studies have tried to ameliorate oxidative injury especially following ischemic and reoxygenation injury [38]. Here, we present the transcriptome of transgenically enhanced Nrf2 (mNrf2-TG), which we previously demonstrated as a precondition to protect the heart against oxidative insults [20]. Enhancing Nrf2 expression in the heart under canonical Keap1 interaction prevents oxidative stress in the myocardium of TG mice expressing full-length Nrf2 (mNrf2-TG) following administration of isoproterenol [20]. On the other hand, the constitutive activation of Nrf2 (truncated Nrf2/CaNrf2-TG) results in a hyper-reductive stress, which induces pathological cardiac remodeling with diastolic dysfunction leading to significant mortality [19]. Based on our previous findings, we hypothesized that the enhanced Keap1-Nrf2 system (mNrf2-TG) uplifts the transcriptional activation of ARE/EpRE genes at moderate levels to escalate the basal defense mechanisms in the heart without inducing quantifiable pathological signaling.

Nrf2 regulates the transcription of >250 genes directly [1,7,9,39,40]. In this cardiac-specific mouse model of full-length Nrf2 (wild-type) expression, through NGS-based RNA sequencing, we observed a multitude of changes in the gene coding for antioxidants and ROS detoxifying enzymes. RNAseq detected 14,665 genes in the mNrf2-TG cardiac transcriptome and 680 of them were (4.6% of total genes) were differentially expressed (*p* < 0.05). Of note, 1031 genes were reported to be differentially expressed in the hearts of mice expressing a truncated version of Nrf2 (CaNrf2-TG) mice [41]. In addition to the altered myocardial transcriptome, we identified that constitutive activation of Nrf2 (CaNrf2) led to a hyper-reductive state (i.e., reductive stress) in the myocardium, which caused pathological cardiac remodeling with diastolic dysfunction and mortality in CaNrf2-TG mice [19]. Although most of the DEGs involved in glutathione metabolism were upregulated in both TGs, the number of genes in a given pathway and the degree of change (up- or downregulation of genes) is significantly different in a dose-dependent manner between the mNrf2-TG (full-length Nrf2) and CaNrf2 TG (truncated Nrf2/constitutively active Nrf2) mice. Interestingly, the pathway analysis of the mNrf2-TG myocardial transcriptome did not appear to disrupt biological pathways including apoptosis, metabolism, mitochondrial energetics, or cell cycle, which is significantly different from our previous observations demonstrating a chronic antioxidant signaling/RS inducing pathological cardiac myopathy [41]. This demonstrates that the antioxidant defense of the cardiomyocytes is increased in mNrf2-TG mouse hearts, without pathological consequences for the heart.

**Figure 5:**
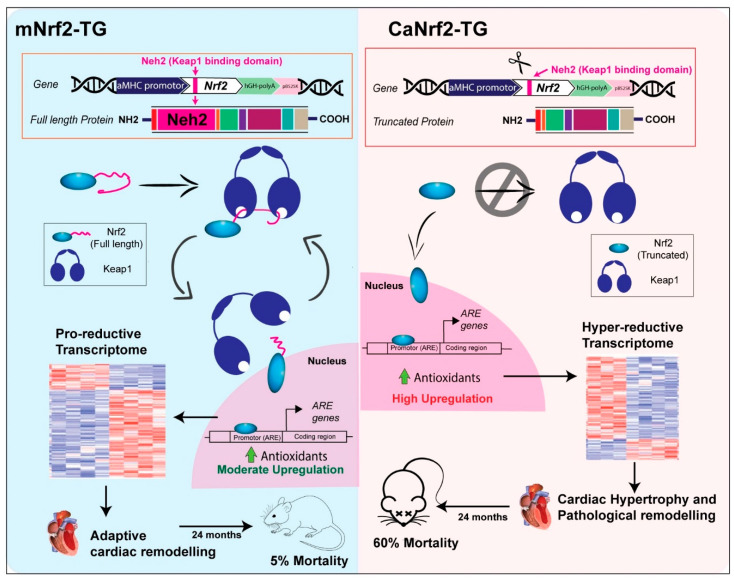
Enhanced Nrf2-Keap1 signalling generates excess Nrf2 at baseline without affecting keap-1 interaction, results in moderate antioxidant upregulation leading to a pro-reductive transcriptome. This leads to an adaptive cardiac remodelling without affecting survival in mNrf2-TG mice. Whereas, affecting the Nrf2-Keap1 signalling renders Nrf2 to be constitutively active. This results in high upregulation of antioxidants that consequentially affect cardiac structure and function resulting in increased mortality rate at 6 months in mice.

The survival of mNrf2-TG exhibiting a pro-reductive redox was unaffected while there was significant mortality seen in hyper-reductive mice. These findings reveal that the glutathione redox state (redox ratio) above 80 is pathological, whereas a redox ratio ranging from 25 to 60 seems to be either non-pathological or less harmful to the myocardium. A distinction between the normal redox (NTG), pro-reductive redox (mNrf2-TG) and hyper-reductive (or reductive stress) redox (CaNrf2-TG) conditions, suggests unique changes in response to a given redox makeup in the myocardium. Overall, these results demonstrate the significance of a mild to moderate activation of transcriptome and a slightly increased biochemical redox makeup are vital for the myocardium to overcome the instant oxidative challenges (Figure 5). Nonetheless, the abundant activation of the Nrf2/ARE-dependent antioxidant system will impair the myocardial health through reductive stress as was evident in the CaNrf2-TG mice [19,27].

Interestingly, we did not notice pathological cardiac hypertrophy or diastolic dysfunction in association with the less robustly activated transcriptome in mNrf2-TG mice, suggesting that the expression of full-length Nrf2 is tightly under the control of its repressor, Keap1. The observed increase in EF, LV mass, IVSd and IVSs in mNrf2-TG hearts might be attributed to a compensatory response to redox changes, as there was no significant mortality noticed in these mice when compared to either NTG or CaNrf2-TG. Cardiac hypertrophy activates fetal gene expression (Nppa, Nppb) among others (HDACs, GATAs) during remodeling [23]. Altered expression in the hypertrophy marker genes in mNrf2-TG indicates that the heart is gradually remodeling when compared to the severe/irreversible pathological remodeling in the CaNrf2-TG/RS hearts (Figure 4E,F). Collectively, our data demonstrate a cardiac tissue-specific augmentation of antioxidant transcriptome assembly, which was previously shown to protect the heart against oxidative insults [18]. Nonetheless, the molecular mechanisms of RS-mediated impairment of structural integrity of cardiomyocytes/cardiac tissues are yet to be investigated.

## 5. Conclusions

NGS and GSEA enrichment revealed that enhanced Keap1-Nrf2 signaling in the myocardium promoted hundreds of ARE and downstream targets, even under unstressed conditions. While constitutively activated Nrf2 is detrimental to the heart, the transgenic expression of wild type Nrf2 could prime the transcriptome and trigger adaptive changes in the heart through a moderately increased antioxidant system.

Future studies will be focused on testing enhanced Keap1-Nrf2 signaling through various stresses but not limited to pharmaceutical agents or exercise using knockout and transgenic methods in vitro and/or in vivo models. One limitation of our study is that only male mice were used for sequencing; however, qPCR validation has been performed in both male and female.

## Data Availability

All data generated for this study are provided as open access on NCBI Gene Expression Omnibus (GEO) accession: GSE202298.

## Supplementary Materials

The following supporting information can be downloaded at: https://www.mdpi.com/article/10.3390/genes13091514/s1, Figure S1: Quality control of RNA-Seq dataset and congruence of the biological samples, Figure S2: Extended pathway analysis. Table S1: Enrichment table of Wikipathways Nrf2 Network; Table S2: Enrichment table of Hallmark’s reactive oxygen species pathway; Table S3: Enrichment table of Reactome Nrf2 glutathione conjugation Table S4: Complete list of real-time qPCR primer sequences and list of primary antibodies.
