# Supplementary material for: Transgenic Expression of Nrf2 Induces a Pro-Reductive Stress and Adaptive Cardiac Remodeling in the Mouse
Source: Genes (Basel). 2022 Aug 24;13(9):1514. doi: 10.3390/genes13091514 (PMC9498410; doi:10.3390/genes13091514)
Supplement: Supplementary file 1 [file genes-13-01514-s001.zip › genes-1732253-supplementary.pdf]

Supplemental Figure S1

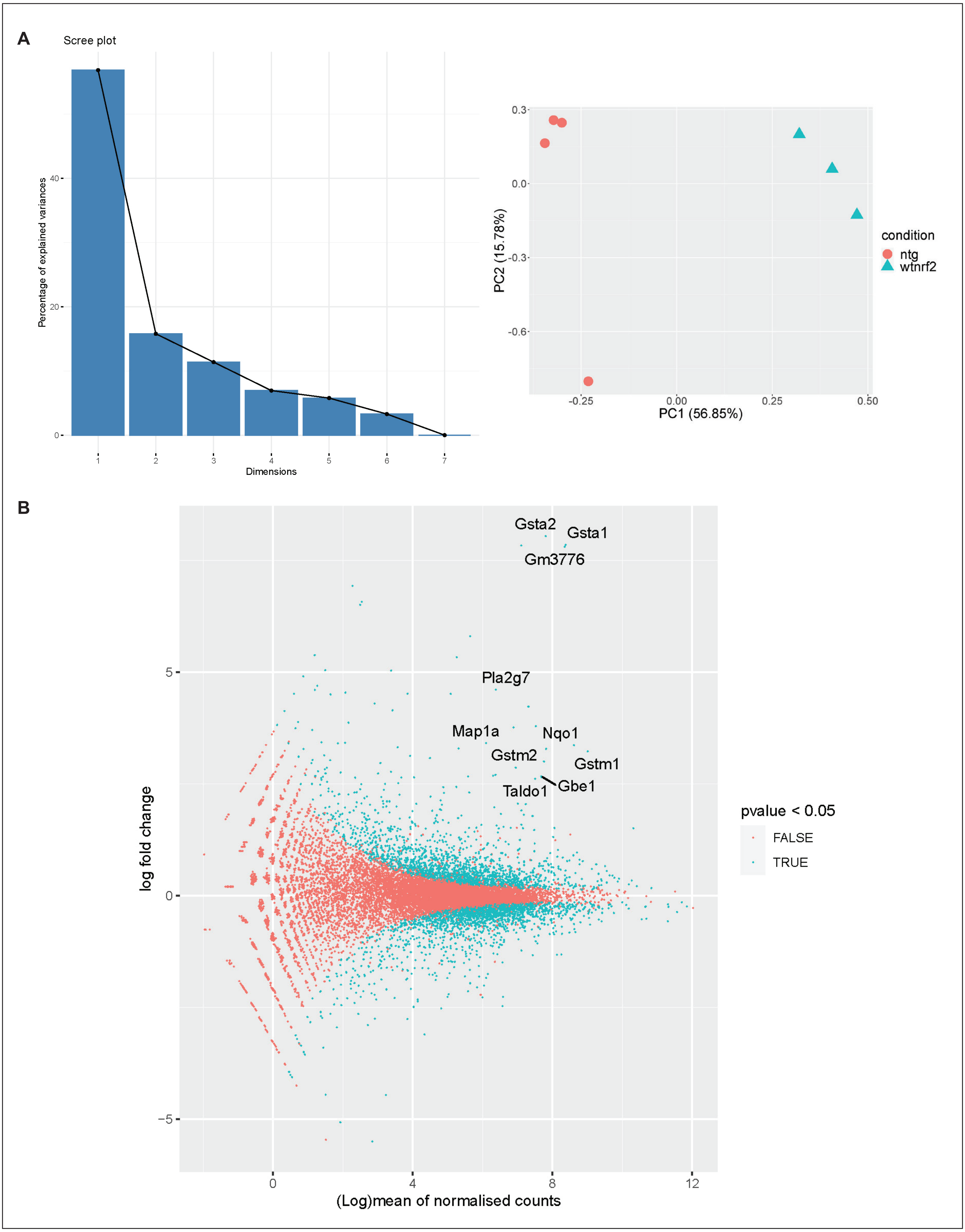

Supplemental Figure S1: Quality control of RNA-Seq dataset and congruence of the biological samples. A The optimal number of components were determined from scree plot. Y axis shows percentage variance each principal component (x axis) captured from the data **B** The first 2 principal components of the samples were plotted in 2-D graph. **C** Gene clusters in multidimensional plot shows the log fold change versus average expression of each gene in mNrf2-TG mice. Blue dots represent significant genes. Top 10 genes with the smallest p-value are labelled.

Supplemental Figure S2:

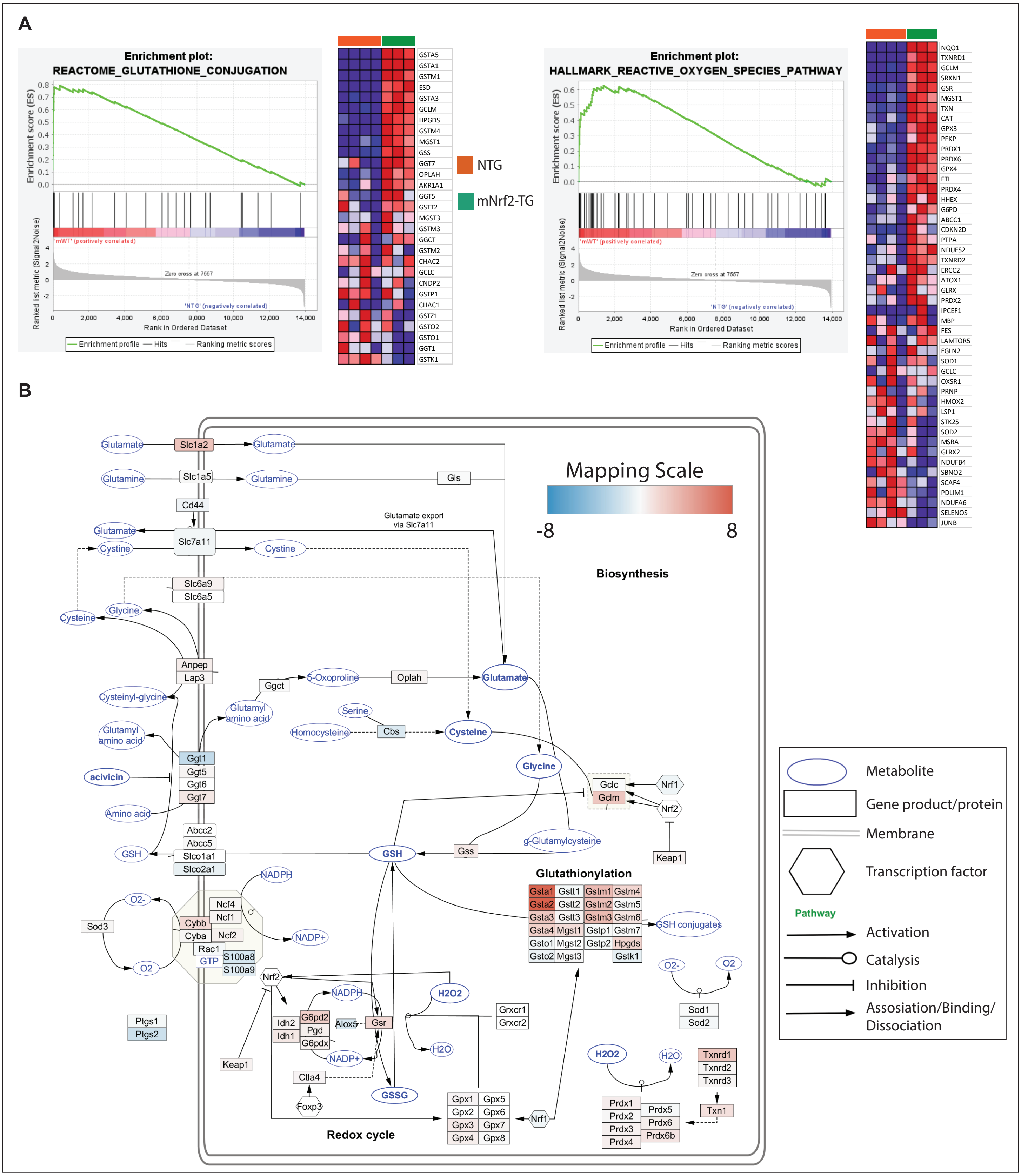

Supplemental Figure S2: Extended pathway analysis. A Enrichment plot and heatmap of gene expression for Reactive oxygen species pathway from Hallmark Band Glutathione conjugation pathway from reactome. **B** Overview of changes occurring in the Oxidative stress and redox pathway were mapped on wikipathways network (WP4466) using Cytoscape for visualization. Nodes represent proteins metabolites or small molecules, Edges (or lines) represent interactions and relationships between connected nodes. Metabolites (such as O2, H2O2, GSH etc) are represented in dark red. Enriched genes are scaled from red to blue, using Log2fold-change. Red represents upregulated genes and blue represents downregulated genes, and white indicates no change. B Validation of protein expression using western blot confirming the summary of changes in gene expression.

Supplemental Table S1:

	PROBE	DESCRIPTION (from dataset)	RANK IN GENE LIST	RANK METRIC SCORE	RUNNING ES	CORE ENRICHMENT
1	GSTA5	glutathione S-transferase alpha 5 [Source:HGNC Symbol;Acc:HGNC:19662]	0	4.959	0.0473	Yes
2	GSTA1	glutathione S-transferase alpha 1 [Source:HGNC Symbol;Acc:HGNC:4626]	2	4.567	0.0909	Yes
3	NQO1	NAD(P)H quinone dehydrogenase 1 [Source:HGNC Symbol;Acc:HGNC:2874]	8	4.095	0.1296	Yes
4	GSTM1	glutathione S-transferase mu 1 [Source:HGNC Symbol;Acc:HGNC:4632]	9	4.063	0.1684	Yes
5	NFE2L2	"nuclear factor, erythroid 2 like 2 [Source:HGNC Symbol;Acc:HGNC:7782]"	10	4.038	0.2070	Yes
6	TXNRD1	thioredoxin reductase 1 [Source:HGNC Symbol;Acc:HGNC:12437]	16	3.610	0.2411	Yes
7	GSTA3	glutathione S-transferase alpha 3 [Source:HGNC Symbol;Acc:HGNC:4628]	17	3.591	0.2754	Yes
8	GCLM	glutamate-cysteine ligase modifier subunit [Source:HGNC Symbol;Acc:HGNC:4312]	18	3.573	0.3095	Yes
9	BLVRB	biliverdin reductase B [Source:HGNC Symbol;Acc:HGNC:1063]	23	3.356	0.3412	Yes
10	GSTM4	glutathione S-transferase mu 4 [Source:HGNC Symbol;Acc:HGNC:4636]	46	2.742	0.3658	Yes
11	SRXN1	sulfiredoxin 1 [Source:HGNC Symbol;Acc:HGNC:16132]	49	2.706	0.3915	Yes
12	GSR	glutathione-disulfide reductase [Source:HGNC Symbol;Acc:HGNC:4623]	56	2.525	0.4152	Yes
13	TXN	thioredoxin [Source:HGNC Symbol;Acc:HGNC:12435]	126	2.031	0.4296	Yes
14	SLC2A9	solute carrier family 2 member 9 [Source:HGNC Symbol;Acc:HGNC:13446]	156	1.874	0.4454	Yes
15	MAFG	MAF bZIP transcription factor G [Source:HGNC Symbol;Acc:HGNC:6781]	385	1.400	0.4423	Yes
16	ABCC3	ATP binding cassette subfamily C member 3 [Source:HGNC Symbol;Acc:HGNC:54]	433	1.340	0.4517	Yes
17	PTGR1	prostaglandin reductase 1 [Source:HGNC Symbol;Acc:HGNC:18429]	439	1.335	0.4641	Yes
18	GPX3	glutathione peroxidase 3 [Source:HGNC Symbol;Acc:HGNC:4555]	446	1.320	0.4762	Yes
19	SLC6A19	solute carrier family 6 member 19 [Source:HGNC Symbol;Acc:HGNC:27960]	458	1.315	0.4880	Yes
20	SLC6A8	solute carrier family 6 member 8 [Source:HGNC Symbol;Acc:HGNC:11055]	514	1.252	0.4960	Yes
21	SLC6A6	solute carrier family 6 member 6 [Source:HGNC Symbol;Acc:HGNC:11052]	558	1.211	0.5044	Yes
22	ALDH3A1	aldehyde dehydrogenase 3 family member A1 [Source:HGNC Symbol;Acc:HGNC:405]	623	1.175	0.5110	Yes
23	CES2	carboxylesterase 2 [Source:HGNC Symbol;Acc:HGNC:1864]	657	1.152	0.5196	Yes
24	PRDX1	peroxiredoxin 1 [Source:HGNC Symbol;Acc:HGNC:9352]	680	1.142	0.5289	Yes
25	SLC39A11	solute carrier family 39 member 11 [Source:HGNC Symbol;Acc:HGNC:14463]	725	1.112	0.5364	Yes
26	PRDX6	peroxiredoxin 6 [Source:HGNC Symbol;Acc:HGNC:16753]	733	1.106	0.5464	Yes
27	SLC2A1	solute carrier family 2 member 1 [Source:HGNC Symbol;Acc:HGNC:11005]	758	1.088	0.5551	Yes
28	FTL	ferritin light chain [Source:HGNC Symbol;Acc:HGNC:3999]	788	1.074	0.5632	Yes
29	PGD	phosphogluconate dehydrogenase [Source:HGNC Symbol;Acc:HGNC:8891]	895	1.013	0.5653	Yes
30	ME1	malic enzyme 1 [Source:HGNC Symbol;Acc:HGNC:6983]	975	0.979	0.5689	Yes
31	TGFB2	transforming growth factor beta 2 [Source:HGNC Symbol;Acc:HGNC:11768]	984	0.976	0.5776	Yes
32	KEAP1	kelch like ECH associated protein 1 [Source:HGNC Symbol;Acc:HGNC:23177]	1180	0.892	0.5721	Yes
33	G6PD	glucose-6-phosphate dehydrogenase [Source:HGNC Symbol;Acc:HGNC:4057]	1249	0.868	0.5754	Yes
34	SQSTM1	sequestosome 1 [Source:HGNC Symbol;Acc:HGNC:11280]	1377	0.831	0.5742	Yes
35	SLC2A6	solute carrier family 2 member 6 [Source:HGNC Symbol;Acc:HGNC:11011]	1511	0.785	0.5721	Yes
36	CBR3	carbonyl reductase 3 [Source:HGNC Symbol;Acc:HGNC:1549]	1575	0.768	0.5748	Yes
37	FTH1	ferritin heavy chain 1 [Source:HGNC Symbol;Acc:HGNC:3976]	1640	0.749	0.5774	Yes
38	SLC2A8	solute carrier family 2 member 8 [Source:HGNC Symbol;Acc:HGNC:13812]	1787	0.708	0.5736	Yes
39	SLC2A13	solute carrier family 2 member 13 [Source:HGNC Symbol;Acc:HGNC:15956]	1790	0.707	0.5802	Yes
40	CBR1	carbonyl reductase 1 [Source:HGNC Symbol;Acc:HGNC:1548]	1797	0.706	0.5865	Yes
41	SLC6A9	solute carrier family 6 member 9 [Source:HGNC Symbol;Acc:HGNC:11056]	1890	0.683	0.5864	Yes
42	SLC6A13	solute carrier family 6 member 13 [Source:HGNC Symbol;Acc:HGNC:11046]	1941	0.674	0.5892	Yes
43	SLC39A12	solute carrier family 39 member 12 [Source:HGNC Symbol;Acc:HGNC:20860]	1942	0.674	0.5956	Yes
44	TGFA	transforming growth factor alpha [Source:HGNC Symbol;Acc:HGNC:11765]	2000	0.663	0.5978	Yes
45	SLC39A13	solute carrier family 39 member 13 [Source:HGNC Symbol;Acc:HGNC:20859]	2071	0.651	0.5990	Yes
46	TXNRD3	thioredoxin reductase 3 [Source:HGNC Symbol;Acc:HGNC:20667]	2236	0.620	0.5930	No
47	SLC6A17	solute carrier family 6 member 17 [Source:HGNC Symbol;Acc:HGNC:31399]	2642	0.545	0.5690	No
48	ABCC4	ATP binding cassette subfamily C member 4 [Source:HGNC Symbol;Acc:HGNC:55]	2898	0.507	0.5554	No
49	SLC39A9	solute carrier family 39 member 9 [Source:HGNC Symbol;Acc:HGNC:20182]	2900	0.507	0.5601	No
50	UGT1A6	UDP glucuronosyltransferase family 1 member A6 [Source:HGNC Symbol;Acc:HGNC:12538]	2953	0.500	0.5611	No
51	SOD3	superoxide dismutase 3 [Source:HGNC Symbol;Acc:HGNC:11181]	3239	0.458	0.5449	No
52	GSTT2	glutathione S-transferase theta 2 (gene/pseudogene) [Source:HGNC Symbol;Acc:HGNC:4642]	3423	0.432	0.5358	No
53	RXRA	retinoid X receptor alpha [Source:HGNC Symbol;Acc:HGNC:10477]	3801	0.382	0.5122	No
54	SLC2A10	solute carrier family 2 member 10 [Source:HGNC Symbol;Acc:HGNC:13444]	3992	0.356	0.5019	No
55	MGST3	microsomal glutathione S-transferase 3 [Source:HGNC Symbol;Acc:HGNC:7064]	4065	0.348	0.5000	No
56	EGR1	early growth response 1 [Source:HGNC Symbol;Acc:HGNC:3238]	4118	0.341	0.4995	No
57	CES3	carboxylesterase 3 [Source:HGNC Symbol;Acc:HGNC:1865]	4224	0.329	0.4950	No
58	AGER	advanced glycosylation end-product specific receptor [Source:HGNC Symbol;Acc:HGNC:320]	4329	0.317	0.4905	No
59	SLC5A6	solute carrier family 5 member 6 [Source:HGNC Symbol;Acc:HGNC:11041]	4563	0.292	0.4765	No
60	SLC39A7	solute carrier family 39 member 7 [Source:HGNC Symbol;Acc:HGNC:4927]	4767	0.267	0.4643	No
61	GSTM3	glutathione S-transferase mu 3 [Source:HGNC Symbol;Acc:HGNC:4635]	5148	0.232	0.4391	No
62	SLC39A3	solute carrier family 39 member 3 [Source:HGNC Symbol;Acc:HGNC:17128]	5225	0.223	0.4357	No
63	ABCC5	ATP binding cassette subfamily C member 5 [Source:HGNC Symbol;Acc:HGNC:56]	5706	0.175	0.4027	No
64	GSTM2	glutathione S-transferase mu 2 [Source:HGNC Symbol;Acc:HGNC:4634]	6387	0.109	0.3546	No
65	SERPINA1	serpin family A member 1 [Source:HGNC Symbol;Acc:HGNC:8941]	6806	0.069	0.3250	No
66	SLC2A2	solute carrier family 2 member 2 [Source:HGNC Symbol;Acc:HGNC:11006]	7204	0.035	0.2967	No
67	NRG1	neuregulin 1 [Source:HGNC Symbol;Acc:HGNC:7997]	7241	0.031	0.2944	No
68	GCLC	glutamate-cysteine ligase catalytic subunit [Source:HGNC Symbol;Acc:HGNC:4311]	7293	0.027	0.2909	No
69	SLC39A5	solute carrier family 39 member 5 [Source:HGNC Symbol;Acc:HGNC:20502]	7563	0.000	0.2715	No
70	PPARD	peroxisome proliferator activated receptor delta [Source:HGNC Symbol;Acc:HGNC:9235]	7886	-0.026	0.2485	No
71	CYP4A11	cytochrome P450 family 4 subfamily A member 11 [Source:HGNC Symbol;Acc:HGNC:2642]	8034	-0.039	0.2382	No
72	CES5A	carboxylesterase 5A [Source:HGNC Symbol;Acc:HGNC:26459]	8213	-0.054	0.2259	No
73	SLC39A1	solute carrier family 39 member 1 [Source:HGNC Symbol;Acc:HGNC:12876]	8464	-0.079	0.2085	No
74	GSTP1	glutathione S-transferase pi 1 [Source:HGNC Symbol;Acc:HGNC:4638]	8469	-0.079	0.2090	No
75	CES1	carboxylesterase 1 [Source:HGNC Symbol;Acc:HGNC:1863]	8590	-0.091	0.2012	No
76	SLC39A6	solute carrier family 39 member 6 [Source:HGNC Symbol;Acc:HGNC:18607]	8860	-0.119	0.1829	No
77	SLC39A10	solute carrier family 39 member 10 [Source:HGNC Symbol;Acc:HGNC:20861]	9223	-0.157	0.1582	No
78	HGF	hepatocyte growth factor [Source:HGNC Symbol;Acc:HGNC:4893]	9256	-0.160	0.1574	No
79	PDGFB	platelet derived growth factor subunit B [Source:HGNC Symbol;Acc:HGNC:8800]	9514	-0.185	0.1406	No
80	SLC2A4	solute carrier family 2 member 4 [Source:HGNC Symbol;Acc:HGNC:11009]	9674	-0.202	0.1310	No
81	HMOX1	heme oxygenase 1 [Source:HGNC Symbol;Acc:HGNC:5013]	10282	-0.265	0.0897	No
82	HBEGF	heparin binding EGF like growth factor [Source:HGNC Symbol;Acc:HGNC:3059]	11077	-0.361	0.0357	No
83	TGFBR2	transforming growth factor beta receptor 2 [Source:HGNC Symbol;Acc:HGNC:11773]	11204	-0.377	0.0302	No

Supplemental Table S1: Enrichment table of Wikipathways Nrf2 Network. Profile of the Running ES Score & Positions of GeneSet Members on the Rank Ordered List

Supplemental Table S1 (Contd).:

	PROBE	DESCRIPTION (from dataset)	RANK IN GENE LIST	RANK METRIC SCORE	RUNNING ES	CORE ENRICHMENT
84	SLC39A2	solute carrier family 39 member 2 [Source:HGNC Symbol;Acc:HGNC:17127]	11294	-0.387	0.0275	No
85	SLC7A11	solute carrier family 7 member 11 [Source:HGNC Symbol;Acc:HGNC:11059]	11502	-0.420	0.0165	No
86	SLC39A14	solute carrier family 39 member 14 [Source:HGNC Symbol;Acc:HGNC:20858]	11642	-0.441	0.0107	No
87	SLC6A4	solute carrier family 6 member 4 [Source:HGNC Symbol;Acc:HGNC:11050]	11959	-0.487	-0.0075	No
88	GGT1	gamma-glutamyltransferase 1 [Source:HGNC Symbol;Acc:HGNC:4250]	12087	-0.507	-0.0118	No
89	SLC39A8	solute carrier family 39 member 8 [Source:HGNC Symbol;Acc:HGNC:20862]	12398	-0.572	-0.0288	No
90	HSP90AB1	heat shock protein 90 alpha family class B member 1 [Source:HGNC Symbol;Acc:HGNC:5258]	12556	-0.610	-0.0343	No
91	SLC2A12	solute carrier family 2 member 12 [Source:HGNC Symbol;Acc:HGNC:18067]	12838	-0.681	-0.0481	No
92	HSP90AA1	heat shock protein 90 alpha family class A member 1 [Source:HGNC Symbol;Acc:HGNC:5253]	12874	-0.696	-0.0440	No
93	FGF13	fibroblast growth factor 13 [Source:HGNC Symbol;Acc:HGNC:3670]	12933	-0.717	-0.0414	No
94	DNAJB1	DnaJ heat shock protein family (Hsp40) member B1 [Source:HGNC Symbol;Acc:HGNC:5270]	13163	-0.812	-0.0502	No
95	EPHA2	EPH receptor A2 [Source:HGNC Symbol;Acc:HGNC:3386]	13211	-0.830	-0.0456	No
96	TGFB1	transforming growth factor beta 1 [Source:HGNC Symbol;Acc:HGNC:11766]	13298	-0.865	-0.0436	No
97	SLC5A3	solute carrier family 5 member 3 [Source:HGNC Symbol;Acc:HGNC:11038]	13356	-0.901	-0.0391	No
98	SLC6A20	solute carrier family 6 member 20 [Source:HGNC Symbol;Acc:HGNC:30927]	13374	-0.917	-0.0316	No
99	SLC2A3	solute carrier family 2 member 3 [Source:HGNC Symbol;Acc:HGNC:11007]	13496	-0.997	-0.0308	No
100	HSPA1A	heat shock protein family A (Hsp70) member 1A [Source:HGNC Symbol;Acc:HGNC:5232]	13511	-1.007	-0.0222	No
101	SLC2A5	solute carrier family 2 member 5 [Source:HGNC Symbol;Acc:HGNC:11010]	13616	-1.091	-0.0193	No
102	MAFF	MAF bZIP transcription factor F [Source:HGNC Symbol;Acc:HGNC:6780]	13888	-1.901	-0.0208	No
103	SLC5A1	solute carrier family 5 member 1 [Source:HGNC Symbol;Acc:HGNC:11036]	13924	-2.522	0.0008	No

Supplemental Table S2:

	SYMBOL	TITLE	RANK IN GENE LIST	RANK METRIC SCORE	RUNNING ES	CORE ENRICHMENT
1	NQO1	NAD(P)H quinone dehydrogenase 1 [Source:HGNC Symbol;Acc:HGNC:2874]	8	4.095	0.0895	Yes
2	TXNRD1	thioredoxin reductase 1 [Source:HGNC Symbol;Acc:HGNC:12437]	16	3.610	0.1684	Yes
3	GCLM	glutamate-cysteine ligase modifier subunit [Source:HGNC Symbol;Acc:HGNC:4312]	18	3.573	0.2469	Yes
4	SRXN1	sulfiredoxin 1 [Source:HGNC Symbol;Acc:HGNC:16132]	49	2.706	0.3043	Yes
5	GSR	glutathione-disulfide reductase [Source:HGNC Symbol;Acc:HGNC:4623]	56	2.525	0.3594	Yes
6	MGST1	microsomal glutathione S-transferase 1 [Source:HGNC Symbol;Acc:HGNC:7061]	84	2.230	0.4066	Yes
7	TXN	thioredoxin [Source:HGNC Symbol;Acc:HGNC:12435]	126	2.031	0.4483	Yes
8	CAT	catalase [Source:HGNC Symbol;Acc:HGNC:1516]	326	1.485	0.4666	Yes
9	GPX3	glutathione peroxidase 3 [Source:HGNC Symbol;Acc:HGNC:4555]	446	1.320	0.4871	Yes
10	PFKP	"phosphofructokinase, platelet [Source:HGNC Symbol;Acc:HGNC:8878]"	580	1.198	0.5039	Yes
11	PRDX1	peroxiredoxin 1 [Source:HGNC Symbol;Acc:HGNC:9352]	680	1.142	0.5219	Yes
12	PRDX6	peroxiredoxin 6 [Source:HGNC Symbol;Acc:HGNC:16753]	733	1.106	0.5425	Yes
13	GPX4	glutathione peroxidase 4 [Source:HGNC Symbol;Acc:HGNC:4556]	748	1.094	0.5655	Yes
14	FTL	ferritin light chain [Source:HGNC Symbol;Acc:HGNC:3999]	788	1.074	0.5863	Yes
15	PRDX4	peroxiredoxin 4 [Source:HGNC Symbol;Acc:HGNC:17169]	834	1.054	0.6063	Yes
16	HHEX	hematopoietically expressed homeobox [Source:HGNC Symbol;Acc:HGNC:4901]	1019	0.957	0.6141	Yes
17	G6PD	glucose-6-phosphate dehydrogenase [Source:HGNC Symbol;Acc:HGNC:4057]	1249	0.868	0.6167	Yes
18	ABCC1	ATP binding cassette subfamily C member 1 [Source:HGNC Symbol;Acc:HGNC:51]	1372	0.832	0.6262	Yes
19	CDKN2D	cyclin dependent kinase inhibitor 2D [Source:HGNC Symbol;Acc:HGNC:1790]	2016	0.660	0.5944	No
20	PTPA	protein phosphatase 2 phosphatase activator [Source:HGNC Symbol;Acc:HGNC:9308]	2204	0.627	0.5948	No
21	NDUFS2	NADH:ubiquinone oxidoreductase core subunit S2 [Source:HGNC Symbol;Acc:HGNC:7708]	2206	0.626	0.6085	No
22	TXNRD2	thioredoxin reductase 2 [Source:HGNC Symbol;Acc:HGNC:18155]	2245	0.617	0.6193	No
23	ERCC2	"ERCC excision repair 2, TFIIH core complex helicase subunit [Source:HGNC Symbol;Acc:HGNC:3434]"	2656	0.542	0.6017	No
24	ATOX1	antioxidant 1 copper chaperone [Source:HGNC Symbol;Acc:HGNC:798]	2736	0.532	0.6077	No
25	GLRX	glutaredoxin [Source:HGNC Symbol;Acc:HGNC:4330]	3068	0.481	0.5945	No
26	PRDX2	peroxiredoxin 2 [Source:HGNC Symbol;Acc:HGNC:9353]	3577	0.410	0.5669	No
27	IPCEF1	interaction protein for cytohesin exchange factors 1 [Source:HGNC Symbol;Acc:HGNC:21204]	4193	0.332	0.5299	No
28	MBP	myelin basic protein [Source:HGNC Symbol;Acc:HGNC:6925]	4895	0.254	0.4851	No
29	FES	"FES proto-oncogene, tyrosine kinase [Source:HGNC Symbol;Acc:HGNC:3657]"	5971	0.150	0.4109	No
30	LAMTOR5	"late endosomal/lysosomal adaptor, MAPK and MTOR activator 5 [Source:HGNC Symbol;Acc:HGNC:17955]"	6062	0.142	0.4076	No
31	EGLN2	egl-9 family hypoxia inducible factor 2 [Source:HGNC Symbol;Acc:HGNC:14660]	6275	0.122	0.3950	No
32	SOD1	superoxide dismutase 1 [Source:HGNC Symbol;Acc:HGNC:11179]	6912	0.059	0.3505	No
33	GCLC	glutamate-cysteine ligase catalytic subunit [Source:HGNC Symbol;Acc:HGNC:4311]	7293	0.027	0.3237	No
34	OXSR1	oxidative stress responsive kinase 1 [Source:HGNC Symbol;Acc:HGNC:8508]	7555	0.000	0.3050	No
35	PRNP	prion protein [Source:HGNC Symbol;Acc:HGNC:9449]	8264	-0.059	0.2553	No
36	HMOX2	heme oxygenase 2 [Source:HGNC Symbol;Acc:HGNC:5014]	8824	-0.115	0.2176	No
37	LSP1	lymphocyte specific protein 1 [Source:HGNC Symbol;Acc:HGNC:6707]	9170	-0.151	0.1960	No
38	STK25	serine/threonine kinase 25 [Source:HGNC Symbol;Acc:HGNC:11404]	9619	-0.196	0.1681	No
39	SOD2	superoxide dismutase 2 [Source:HGNC Symbol;Acc:HGNC:11180]	10462	-0.283	0.1137	No
40	MSRA	methionine sulfoxide reductase A [Source:HGNC Symbol;Acc:HGNC:7377]	10666	-0.306	0.1058	No
41	GLRX2	glutaredoxin 2 [Source:HGNC Symbol;Acc:HGNC:16065]	10997	-0.349	0.0897	No
42	NDUFB4	NADH:ubiquinone oxidoreductase subunit B4 [Source:HGNC Symbol;Acc:HGNC:7699]	11618	-0.438	0.0547	No
43	SBNO2	strawberry notch homolog 2 [Source:HGNC Symbol;Acc:HGNC:29158]	12233	-0.536	0.0223	No
44	SCAF4	SR-related CTD associated factor 4 [Source:HGNC Symbol;Acc:HGNC:19304]	12593	-0.617	0.0100	No
45	PDLIM1	PDZ and LIM domain 1 [Source:HGNC Symbol;Acc:HGNC:2067]	13099	-0.774	-0.0093	No
46	NDUFA6	NADH:ubiquinone oxidoreductase subunit A6 [Source:HGNC Symbol;Acc:HGNC:7690]	13425	-0.953	-0.0118	No
47	SELENOS	selenoprotein S [Source:HGNC Symbol;Acc:HGNC:30396]	13600	-1.078	-0.0006	No
48	JUNB	"JunB proto-oncogene, AP-1 transcription factor subunit [Source:HGNC Symbol;Acc:HGNC:6205]"	13639	-1.120	0.0213	No

Supplemental Table S2: Enrichment table of HALLMARK's Reactive oxygen species pathway. Profile of the Running ES Score & Positions of GeneSet Members on the Rank Ordered List

Supplemental Table S3:

PROBE	DESCRIPTION (from dataset)	GENE SYMBOL	RANK IN GENE LIST	RANK METRIC SCORE	RUNNING ES	CORE ENRICHMENT
1	GSTA5	glutathione S-transferase alpha 5 [Source:HGNC Symbol;Acc:HGNC:19662]	0	4.959	0.1213	Yes
2	GSTA1	glutathione S-transferase alpha 1 [Source:HGNC Symbol;Acc:HGNC:4626]	2	4.567	0.2329	Yes
3	GSTM1	glutathione S-transferase mu 1 [Source:HGNC Symbol;Acc:HGNC:4632]	9	4.063	0.3319	Yes
4	ESD	esterase D [Source:HGNC Symbol;Acc:HGNC:3465]	15	3.643	0.4207	Yes
5	GSTA3	glutathione S-transferase alpha 3 [Source:HGNC Symbol;Acc:HGNC:4628]	17	3.591	0.5084	Yes
6	GCLM	glutamate-cysteine ligase modifier subunit [Source:HGNC Symbol;Acc:HGNC:4312]	18	3.573	0.5958	Yes
7	HPGDS	hematopoietic prostaglandin D synthase [Source:HGNC Symbol;Acc:HGNC:17890]	44	2.777	0.6620	Yes
8	GSTM4	glutathione S-transferase mu 4 [Source:HGNC Symbol;Acc:HGNC:4636]	46	2.742	0.7290	Yes
9	MGST1	microsomal glutathione S-transferase 1 [Source:HGNC Symbol;Acc:HGNC:7061]	84	2.230	0.7809	Yes
10	GSS	glutathione synthetase [Source:HGNC Symbol;Acc:HGNC:4624]	387	1.397	0.7934	Yes
11	GGT7	gamma-glutamyltransferase 7 [Source:HGNC Symbol;Acc:HGNC:4259]	1119	0.921	0.7633	No
12	OPLAH	"5-oxoprolinase, ATP-hydrolysing [Source:HGNC Symbol;Acc:HGNC:8149]"	1431	0.814	0.7609	No
13	AKR1A1	aldo-keto reductase family 1 member A1 [Source:HGNC Symbol;Acc:HGNC:380]	1670	0.739	0.7618	No
14	GGT5	gamma-glutamyltransferase 5 [Source:HGNC Symbol;Acc:HGNC:4260]	2171	0.634	0.7414	No
15	GSTT2	glutathione S-transferase theta 2 (gene/pseudogene) [Source:HGNC Symbol;Acc:HGNC:4642]	3423	0.432	0.6620	No
16	MGST3	microsomal glutathione S-transferase 3 [Source:HGNC Symbol;Acc:HGNC:7064]	4065	0.348	0.6244	No
17	GSTM3	glutathione S-transferase mu 3 [Source:HGNC Symbol;Acc:HGNC:4635]	5148	0.232	0.5523	No
18	GGCT	gamma-glutamylcyclotransferase [Source:HGNC Symbol;Acc:HGNC:21705]	6051	0.143	0.4909	No
19	GSTM2	glutathione S-transferase mu 2 [Source:HGNC Symbol;Acc:HGNC:4634]	6387	0.109	0.4695	No
20	CHAC2	ChaC glutathione specific gamma-glutamylcyclotransferase 2 [Source:HGNC Symbol;Acc:HGNC:32363]	6837	0.066	0.4389	No
21	GCLC	glutamate-cysteine ligase catalytic subunit [Source:HGNC Symbol;Acc:HGNC:4311]	7293	0.027	0.4068	No
22	CNDP2	carnosine dipeptidase 2 [Source:HGNC Symbol;Acc:HGNC:24437]	7626	-0.002	0.3830	No
23	GSTP1	glutathione S-transferase pi 1 [Source:HGNC Symbol;Acc:HGNC:4638]	8469	-0.079	0.3244	No
24	CHAC1	ChaC glutathione specific gamma-glutamylcyclotransferase 1 [Source:HGNC Symbol;Acc:HGNC:28680]	8719	-0.105	0.3090	No
25	GSTZ1	glutathione S-transferase zeta 1 [Source:HGNC Symbol;Acc:HGNC:4643]	10440	-0.281	0.1922	No
26	GSTO2	glutathione S-transferase omega 2 [Source:HGNC Symbol;Acc:HGNC:23064]	10501	-0.287	0.1949	No
27	GSTO1	glutathione S-transferase omega 1 [Source:HGNC Symbol;Acc:HGNC:13312]	11369	-0.398	0.1423	No
28	GGT1	gamma-glutamyltransferase 1 [Source:HGNC Symbol;Acc:HGNC:4250]	12087	-0.507	0.1032	No
29	GSTK1	glutathione S-transferase kappa 1 [Source:HGNC Symbol;Acc:HGNC:16906]	13711	-1.211	0.0161	No

Supplemental Table S3: Enrichment table of reactome Nrf2 Glutathione conjugation. Profile of the Running ES Score & Positions of GeneSet Members on the Rank Ordered List

Supplemental Table S4A

Gene	Forward	Reverse
Acta2	CCCTGGAGAAGAGCTACGAAC	TTTCGTGGATGCCCGCTG
Arbp1	TGAGATTCGGGATATGCTGTTGG	CGGGTCCTAGACCAGTGTTCT
Gapdh	TGACCTCAACTACATGGTCTACA	CTTCCCATTCTCGGCCTTG
Gclc	GGACAAACCCCAACCATCC	GTTGAACTCAGACATCGTTCCT
Gclm	CTTCGCCTCCGATTGAAGATG	AAAGGCAGTCAAATCTGGTGG
Gsr	CACGGCTATGCAACATTCGC	GTGTGGAGCGGTAAACTTTTTC
Gsta3	AAGAATGGAGCCTATCCGGTG	CCATCACTTCGTAACCTTGCC
Gstm1	GATTGGTGCAGGGTTGGGAG	GCT GGT GCT GTG GTC TTC TC
Gstt2	TGCCCAAGTCCACGAATACC	CCATTCTATCTCTGTTCCGTTCC
Hdac2	GCTTGCCATCCTCGAATTACT	GTC ATC ACG CGATCTGTTGTAT
Hdac4	CTGCAAGTGGCC CCTACA G	CTG CTC ATG TTG ACG CTG GA
Hdac6	GAGGAGCTGATGTTGGTTCAC	AGTTCGGATGCAGATACACTGA
mNrf2-TG	ACTTTACATGGAGTCCTGGTGGGA	TCTCCTGTTCCTTCTGGAGTTGCT
Myh6 (αMHC)	GAGTGGGAGTTTATCGACTTCG	CCTTGACATTGCGAGGCTTC
Myh7	ACTGTCAACACTAAGAGGGTCA	TTGGATGATTTGATCTTCCAGGG
Nppa	GCTTCCAGGCCATATTGGAG	GGGGGCATGACCTCATCTT
Nppb	GAGGTCACTCCTATCCTCTGG	GCCATTTCCTCCGACTTTTCTC
Nqo1	AGGATGGGAGGTACTCGAATC	TGCTAGAGATGACTCGGAAGG
Nrf2	CTGAACTCCTGGACGGGACTA	CGGTGGGTCTCCGTAAATGG
Tgfbr2	CCGCTGCATATCGTCCTGTG	AGTGGATGGATGGTCCTATTACA
Txnrd1	GGGTCCTATGACTTCGACCTG	AGTCGGTGTGACAAAATCCAAG

Supplemental Table S4B

Antibody	Host	Company	Catalogue #
CATALASE	Anti-rabbit	EMD Millipore	219010
GCLM	Anti-rabbit	Abcam	AB81445
GPX1	Anti-rabbit	Abcam	AB22604
GSR	Anti-rabbit	Abcam	AB16801
NOGO	Anti-rabbit	Novusbio	56681
NQO1	Anti-rabbit	Abcam	AB34173
SOD2	Anti-rabbit	Abcam	AB13534

Supplemental Table S4: A Complete list of real-time qPCR primer sequences
B List of Primary Antibodies.
